# Regulatory Mechanisms of Salinity-Induced Triterpenoid Saponin Biosynthesis in *Cyclocarya paliurus* Seedling Revealed by Integrated Multi-Omics Analysis and Molecular Docking

**DOI:** 10.3390/plants15101535

**Published:** 2026-05-18

**Authors:** Kun Hong, Hui Chen, Jian Qin, Shengzuo Fang, Xulan Shang, Lei Zhang

**Affiliations:** 1 State Key Laboratory for Development and Utilization of Forest Food Resources, Nanjing Forestry University, Nanjing 210037, China; 2 College of Forestry and Grassland, Co-Innovation Center for the Sustainable Forestry in Southern China, Nanjing Forestry University, Nanjing 210037, China

**Keywords:** *Cyclocarya paliurus*, triterpenoid saponin biosynthesis, WGCNA, salt stress, molecular docking

## Abstract

Soil salinity is a major environmental constraint limiting plant productivity and modulating secondary metabolism. Triterpenoid saponins play crucial roles in plant stress adaptation, yet their biosynthetic regulation in *Cyclocarya paliurus* under salt stress remains poorly understood. This research integrated transcriptomic and metabolomic analyses to investigate triterpenoid saponin metabolism in *C. paliurus* leaves at four NaCl concentrations and two sampling times. Salt stress altered ion homeostasis, suppressed growth, and induced distinct triterpenoid saponins accumulation patterns, with cyclocaric acid B and oleanolic acid showing significant increases. Weighted gene co-expression network analysis identified two modules significantly correlated with triterpenoid saponin accumulation and highlighted transcription factors including WRKY18, bHLH121, ERF4, and ERF1 as regulators of key biosynthetic genes (*DXS*, *SQS*, and *HMGR*). Molecular docking further validated these regulatory interactions, demonstrating that bHLH35, MYC2, ERF113, and MED26B form stable complexes with target gene promoters through extensive hydrogen-bond networks. These findings elucidate the regulatory framework of triterpenoid saponin metabolism under salinity and provide a foundation for molecular breeding and cultivation of *C. paliurus* in saline regions.

## 1. Introduction

Triterpenoid saponins are glycosides formed by the combination of sapogenins and sugars. Among the triterpenoid compounds discovered to date, tetracyclic and pentacyclic structures are the most common [1]. They are widely distributed in plants as secondary metabolites. Triterpenoid saponins not only play a role in plant signal transduction and stress regulation, but also exert various biological functions in the human body such as anti-inflammatory, anti-tumor, antiviral, antibacterial, antioxidant, and hepatoprotective effects [2,3]. For example, lupeol alleviates oxidative stress in *Brassica nigra* under salt stress [4]. Oleanolic acid and maslinic acid demonstrate potent antitumor efficacy against lung carcinoma by inducing apoptosis [5]. Similarly, asiatic acid ameliorates ischemia/reperfusion-induced liver injury through attenuation of oxidative stress and restoration of mitochondrial function [6]. Given these pharmacological properties, triterpenoid saponins hold substantial potential for applications in human health and represent a growing area of commercial interest [7].

Soil salinity poses a significant constraint to global plant productivity. In Jiangsu Province, China, the salt content of coastal saline surface soil ranges from 0.20% to 0.50% [8]. Prolonged salt stress leads to Na^+^ accumulation, nutrient imbalance, and ion toxicity in plants, which can severely reduce yield and even cause plant mortality. Correspondingly, plants implement physiological and biochemical adjustments to maintain ionic and osmotic homeostasis [9]. Secondary metabolites play key roles in such adaptive responses. For example, triterpenoids are important to maintain membrane integrity and improve performance in saline environments [10]. Flavonoids act as scavengers of reactive oxygen species (ROS) under salt stress [11]. Given the dual importance of triterpenoids as high-value pharmaceutical compounds and essential stress-tolerance factors, investigating their biosynthetic pathways and regulatory networks under salt stress is of paramount importance.

*Cyclocarya paliurus* (Batal) IIjinskaja, commonly referred to as “sweet tea tree”, is the sole extant species of the genus *Cyclocarya* in the Juglandaceae family. It is predominantly distributed in mountainous areas of southern and eastern China [12]. The leaves of *C. paliurus* are rich in triterpenoid saponins, flavonoids and other bioactive components, which have been associated with hypoglycemic, hypotensive, and hypolipidemic effects [13]. Under 0.4% (*m*/*v*) salt concentration, the salt injury index of *C. paliurus* increased while seedling height and chlorophyll fluorescence parameters decreased, indicating that *C. paliurus* is a salt-sensitive plant [14]. To meet growing demand for medicinal *C. paliurus* leaves, cultivation in coastal saline soils has been proposed as a potential land-use strategy. However, the transcriptional regulatory network underlying triterpenoid saponin metabolism in this species under salt stress remains uncharacterized.

Integrated transcriptomic and metabolomic approaches have become valuable tools for elucidating gene regulatory networks and metabolite profiles in plants under environmental stress [15,16]. For instance, combined transcriptomic and metabolomic analysis has been used to uncover regulatory mechanisms of flavonoid metabolism in salt-stressed *C. paliurus* seedlings, offering insights for molecular breeding of salt-tolerant genotypes [17]. The present study aims to (1) investigate the effects of sampling time and salt concentration gradients on content and composition of triterpenoid saponins; (2) identify key structural genes and transcription factors (TFs) related to triterpenoid saponin metabolism under salt stress; and (3) construct a regulatory network for triterpenoid saponin metabolism and related gene expression. The findings in this study are anticipated to advance our understanding of molecular regulatory mechanisms underlying enhanced environmental adaptation in plants, while providing theoretical foundations for the sustainable utilization of saline-affected soils in silvicultural practices.

## 2. Results

### 2.1. Variations in Ion Content and Growth Parameters

Salt stress significantly altered the ion content in *C. paliurus* leaves (Table 1). Relative to the control, K^+^ accumulation was significantly elevated in all salt treatments except LS at T_1_. At HS, K^+^ concentrations increased by 13.1% at T_1_ and 15.9% at T_2_ compared to the corresponding CK. In contrast, Ca^2+^ content in the leaves of the salt treatments was significantly lower than that of corresponding CK at both sampling times. The most pronounced reductions were observed under HS, where Ca^2+^ decreased by 27.4% at T_1_ and 32.3% at T_2_ relative to CK. Meanwhile, Na^+^ contents were significantly increased under salt treatments. Under HS, Na^+^ content exhibited a dramatic increase, reaching more than 47-fold that of CK at T_1_ and approximately 15-fold at T_2_. It is noteworthy that Na^+^ content in leaves increased with increasing treatment time, with LS, MS and HS of T_2_ increasing by 89.2%, 238.7% and 152.0%, respectively, compared to T_1_. In a similar fashion to the changes in Ca^2+^ content, the Mg^2+^ content of the plant leaves decreased significantly with increasing salt concentration. Compared to CK, Mg^2+^ was reduced by 28.8% and 37.4% in HS of T_1_ and T_2_, respectively (*p* < 0.05).

**Figure 1:**
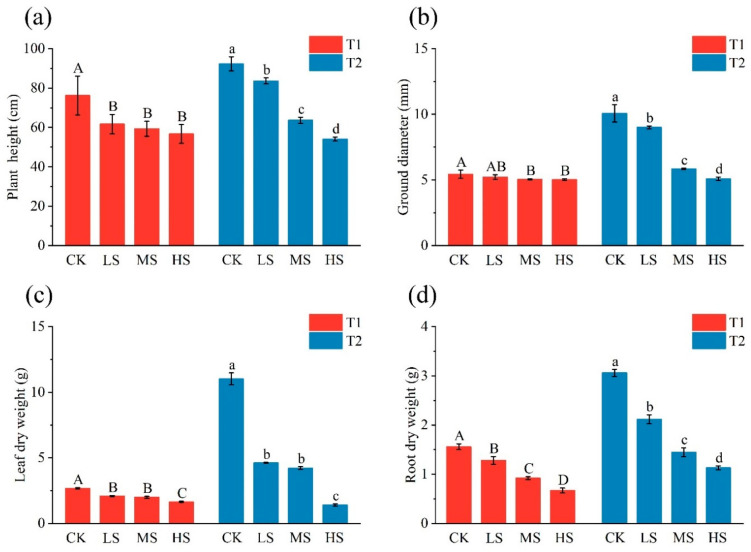
Growth parameters of *C. paliurus* seedlings: (**a**) plant height, (**b**) ground diameter, (**c**) leaf dry weight and (**d**) root dry weight. Capital and lowercase letters indicate significant differences (*p* < 0.05) among treatments at T_1_ and T_2_, respectively. Refer to Table 1 for CK, LS, MS, HS, T_1_ and T_2_.

Compared to CK, salt treatment significantly reduced (*p* < 0.05) the growth parameters of *C. paliurus* (Figure 1). Plant height and root dry weight under salt treatment gradually decreased with increasing salt concentration (Figure 1a,d). On the other hand, the ground diameter and leaf dry weight exhibited a decline in response to increasing salt concentration, albeit this decline was only observed at T_2_ (Figure 1b,c). Interestingly, ground diameter and root dry weight were lower in all groups at T_1_ than at the corresponding T_2_ (Figure 1b,d).

### 2.2. Triterpenoid Saponin Accumulation in C. paliurus Leaves

As shown in Table 2, salt treatment at T_1_ significantly increased the total triterpenoid saponin and major monomer content in *C. paliurus* leaves, while no significant effect was observed at T_2_. Specifically, the levels of cyclocaric acid B and oleanolic acid were significantly elevated under salt stress. Compared with CK, cyclocaric acid B in HS increased by 12.5% and 53.3% at T_1_ and T_2_, respectively, while oleanolic acid increased by 200.0% and 196.0%. In contrast, the content of arjunolic acid exhibited a significant decrease in proportion with increasing salt concentration; at T_1_ and T_2_, its levels in HS were reduced by 23.2% and 75.0% compared to LS, respectively (*p* < 0.05).

### 2.3. Transcriptome Profiles of C. paliurus Under Salt Stress

**Figure 2:**
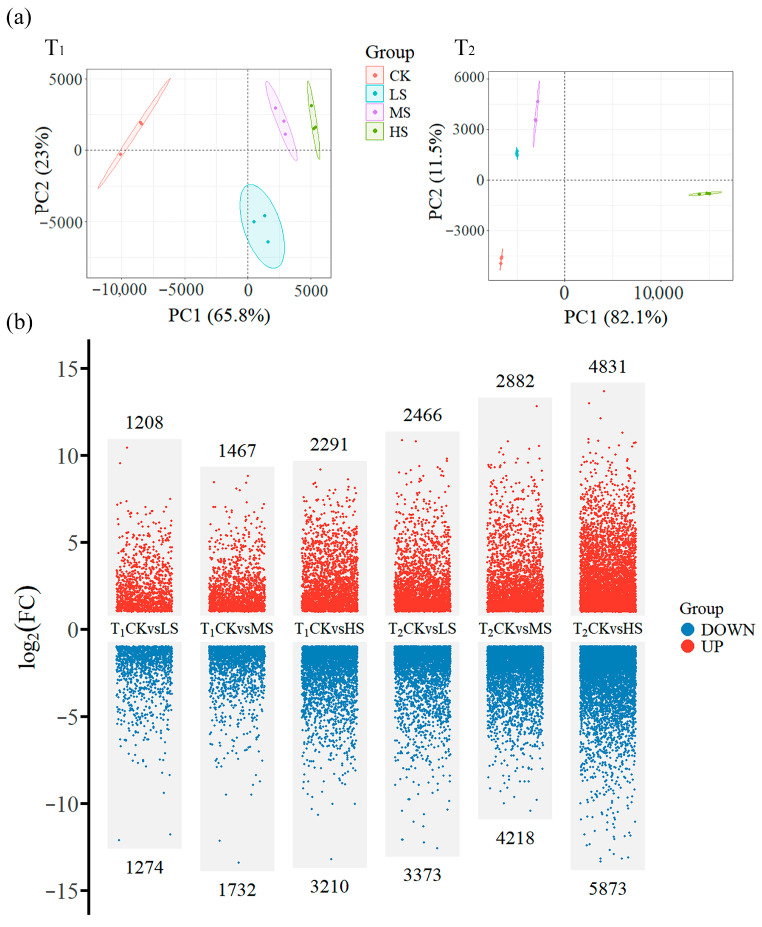
PCA and differential expression analysis of transcripts under various salt treatments. (**a**) PCA score plots of gene expression. Each data point represents an independent biological replicate, with colors (red, blue, purple, and green) denoting CK, LS, MS, and HS groups, respectively. (**b**) Volcano plots of DEGs between the CK and salt-treated groups at T_1_ and T_2_. Differentially expressed genes (DEGs) were identified based on |log_2_FC| > 1 and FDR < 0.05. The numerical values in the figure indicate the total count of DEGs between treatment pairs. Refer to Table 1 for definitions of CK, LS, MS, HS, T_1_, and T_2_.

PCA analyses were performed on the obtained gene expression files in order to elucidate the interrelationships between the different treatments (Figure 2a). The PCA plots for both sampling time showed a clear separation between samples from different treatments, with PC1 explaining 65.8% of the variation between samples for T_1_ and 82.1% for T_2_. This finding suggests that significant transcriptional reprogramming occurs in the leaves of *C. paliurus* in response to salt stress. A further comparison of gene expression between CK and different salt treatments revealed that the number of DEGs increased gradually with increasing salt concentration (Figure 2b). Notably, a greater number of DEGs were identified between CK and the various salt treatments at T_2_ compared to T_1_. The highest number of DEGs was observed between CK and HS at T_2_, with 10,704 DEGs (4831 upregulated genes and 5873 downregulated genes), whereas the number of DEGs at T_1_ (2291 upregulated genes and 3210 downregulated genes) was only 5501 (Figure 2b). Log_2_ (FC) values were obtained for the 39 structural genes associated with the triterpenoid biosynthesis pathway. The majority of the structural genes exhibited log_2_ (FC) values less than −1 at T_2_, with the CK and MS. Up to 36 genes were identified as being downregulated between CK and MS (Table A1).

### 2.4. Analysis of Gene Expression Trend

**Figure 3:**
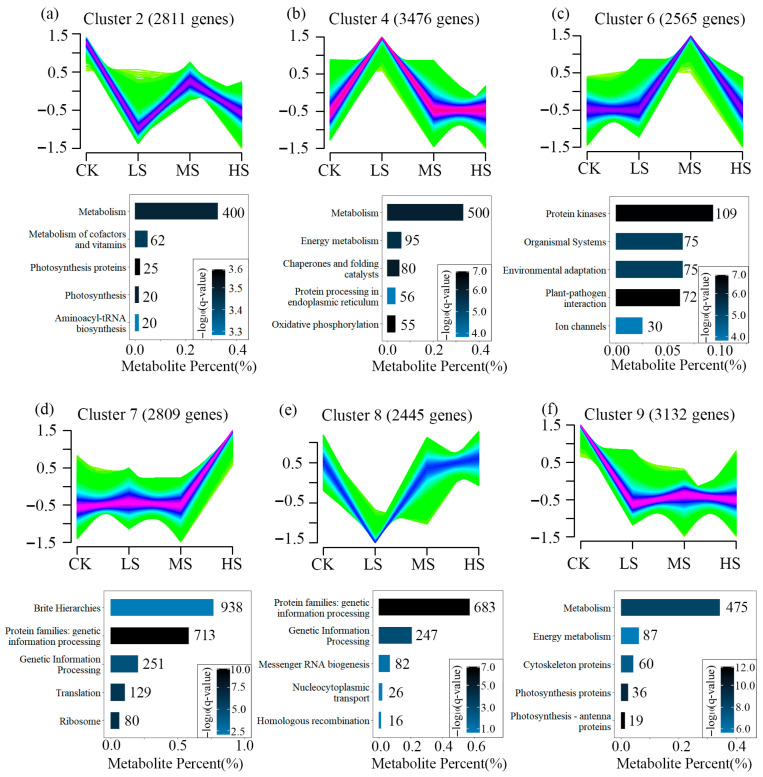
The dynamic expression patterns of genes, which were grouped into six clusters based on the similarity of their abundance profiles (**a**–**f**). Below each cluster, the top five most significantly enriched KEGG pathways; the number on the right side of the bar represents the count of metabolites.

Co-expression profiles of genes co-expressed at four different salt concentrations in T_2_ showed that thousands of genes were categorized into nine different oscillatory patterns, identifying the six most significant salt concentration expression patterns (Figure 3). For each pattern, the five most significantly enriched KEGG pathways were highlighted. A total of 9419 genes from clusters 2, 4, and 9 exhibited a decreasing expression trend with increasing salt concentrations (Figure 3a,b,f), and were primarily associated with photosynthesis and energy metabolism. In contrast, 7819 genes from clusters 6, 7, and 8 showed the opposite trend, with low expression in CK and elevated expression under HS conditions (Figure 3c–e), and were mainly enriched in pathways related to protein metabolism, signal transduction, environmental adaptation, and ion channels.

### 2.5. Metabolomic Response to Salt Stress

**Figure 4:**
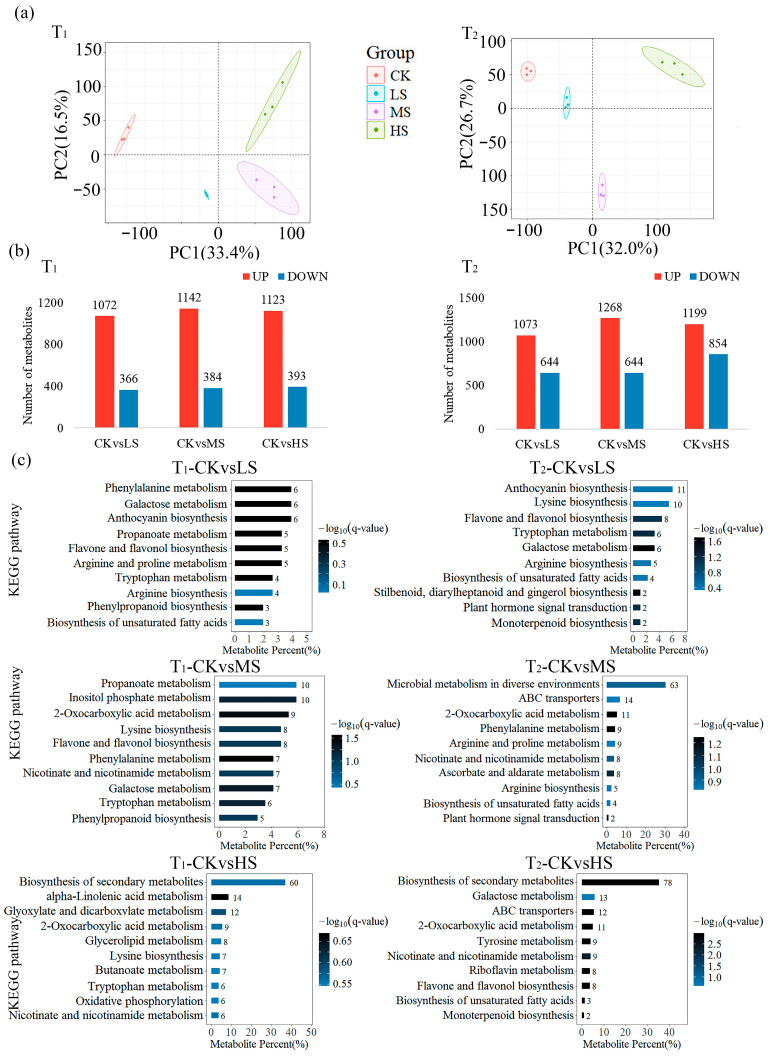
Multivariate and functional enrichment analysis of metabolites and genes in *C. paliurus*. (**a**) PCA score plots of gene expression profiles. Each point represents an independent biological replicate, with colors (red, blue, purple, and green) denoting CK, LS, MS, and HS, respectively. (**b**) Total number of differentially expressed genes (DEGs) and differentially accumulated metabolites (DAMs) identified between treatment pairs at each sampling time. (**c**) KEGG pathway enrichment bar chart of the DAMs between CK and salt-treatment groups. Refer to Table 1 for definitions of CK, LS, MS, HS, T_1_, and T_2_.

PCA analysis of metabolome data from two sampling times under different treatments demonstrated a significant separation between samples from different salt treatments, thereby suggesting that the accumulation of substances at the metabolome level responded significantly to the level of salt stress (Figure 4a). Comparative analysis of metabolome data led to the identification of 24 metabolites belonging to the triterpenoid pathway, the majority of which exhibited significant variations in response to the salt treatments (Table A2). Further comparison of DAMs between CK and different salt treatments at both sampling times revealed that there were more DAMs in T_2_ than in T_1_ (Figure 4b).

In order to enhance the comprehension of the potential functional mechanisms of diverse metabolites in the context of salt stress, KEGG pathway enrichment analysis was conducted in this study (Figure 4c). DAMs were enriched in the biosynthesis of the secondary metabolite pathway between HS and CK at both sampling times, with 60 and 78 metabolites detected, respectively. Furthermore, two metabolites were identified in the monoterpenoid biosynthesis pathway between HS and CK at T_2_.

### 2.6. Screening of Key Genes and Metabolites and Physiological Correlations

**Figure 5:**
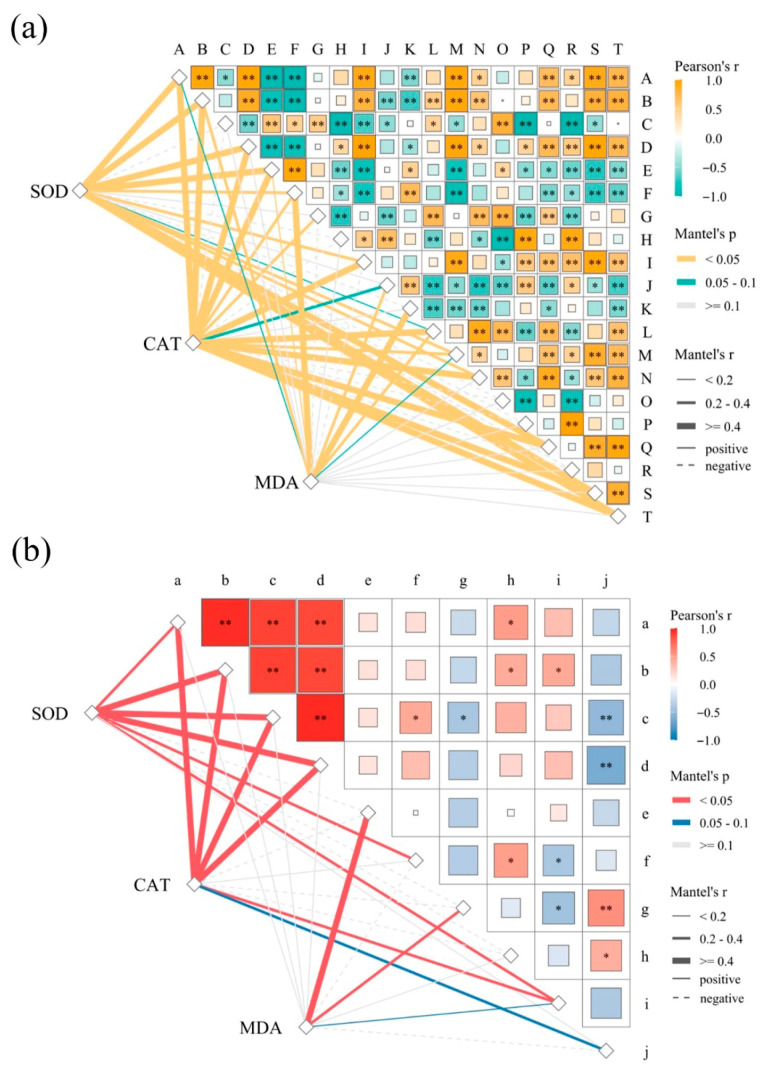
Mantel test visualization showing the correlations between physiological indicators and molecular profiles. (**a**) Relationships between antioxidant enzymes (SOD and CAT), MDA content, and 20 key candidate genes. (**b**) Correlations between physiological indicators and 10 major metabolites. The color scale represents Pearson’s correlation coefficients ranging from −1 to 1; warmer tones indicate positive correlations, while colder tones indicate negative correlations. The overlaying lines (or shaded areas) represent Mantel’s *r* and *p*-values, where line width and shade intensity reflect the statistical significance (Mantel’s * *p* < 0.05 or ** *p* < 0.01). Refer to Table 1 for treatment details.

The physiological status of *C. paliurus* under salt stress was assessed by measuring the activities of superoxide dismutase (SOD), catalase (CAT), and the concentration of malondialdehyde (MDA). Based on statistical significance and descriptions, we selected the top 20 key regulatory genes from the DEGs between CK and HS, including 13 upregulated and 7 downregulated genes (Table A3). The upregulated genes were mainly involved in the regulation of plant defense–related metabolic pathways, phenylpropanoid metabolism, and synthesis of secondary metabolites, such as triterpenoid saponin biosynthesis. In contrast, the downregulated genes were primarily associated with photosynthesis, ATP synthesis, and abscisic acid degradation. A Mantel test heatmap (Figure 5a) showed that genes highly correlated with MDA content were predominantly downregulated, including F (transketolase), J (lycopene epsilon cyclase), and K (caffeic acid 3-O-methyltransferase). Conversely, genes highly correlated with SOD and CAT activities were mostly upregulated, including D (hydroxycinnamoyl transferase), M (β-amyrin synthase), and S (crocetin glucosyltransferase).

Similarly, we identified the top 10 key DAMs between CK and HS, comprising eight upregulated and two downregulated metabolites (Table A4). The upregulated metabolites were mainly involved in phenylpropanoid metabolism, as well as the biosynthesis of various alkaloids and other secondary metabolites, including phenylglyoxylic acid, gallic acid, and benzoic acid. The downregulated metabolites were associated with C5-branched dibasic acid metabolism and galactose metabolism, represented by itaconic acid and galactinol. The Mantel test (Figure 5b) further revealed that high correlation with MDA activity was observed for e (benzoic acid), which was upregulated, whereas g (itaconic acid) was downregulated. Metabolites strongly correlated with SOD and CAT activities, including a (phenylglyoxylic acid), b (gallic acid), c (secoisolariciresinol), and d (D-proline), exhibited significant upregulation. Notably, phenylglyoxylic acid, gallic acid, and secoisolariciresinol were all closely associated with phenylpropanoid metabolism.

### 2.7. Co-Expression Network Analysis of Weighted Genes

**Figure 6:**
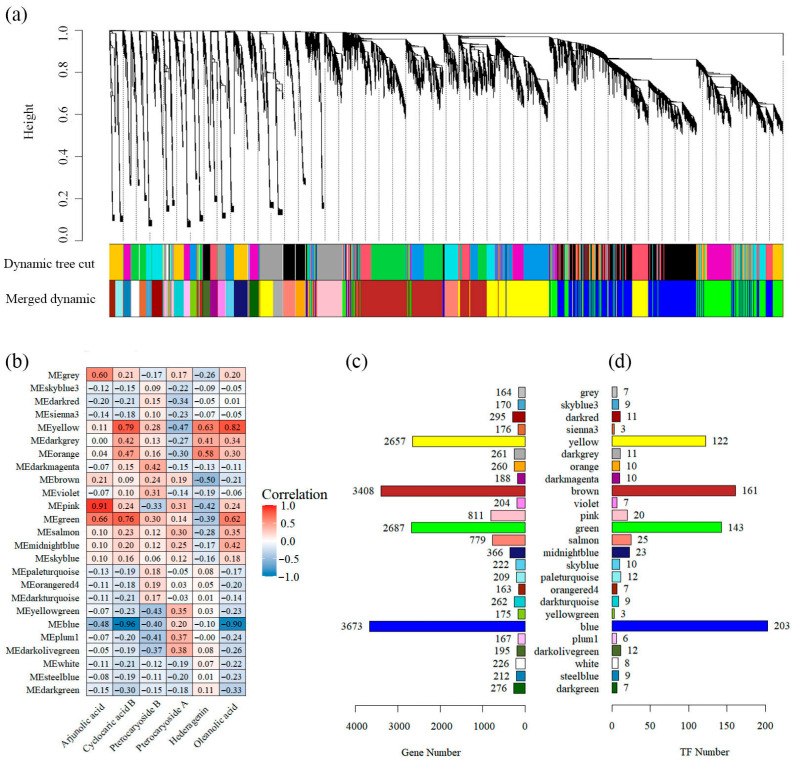
WGCNA identification of co-expression modules associated with amino acid accumulation. (**a**) Hierarchical clustering dendrogram of expressed genes, with identified modules assigned distinct colors. (**b**) Heatmap illustrating the correlations between amino acid contents and module eigengenes (MEs). The color scale represents the correlation coefficient, where red and blue indicate positive and negative correlations, respectively. The number of genes (**c**) and TFs (**d**) contained in each module are shown, with each module represented by rectangles of different colors.

To investigate triterpenoid metabolism in *C. paliurus* leaves, a weighted gene co-expression network analysis (WGCNA) was performed on 18,206 genes (after the filtration of deletion and outlier values), which were clustered into 25 modules (Figure 6a). The modules of six compounds (arjunolic acid, cyclocaric acid B, pterocaryoside B, pterocaryoside A, hederagenin and oleanolic acid) related to triterpenoid metabolism of *C. paliurus* and contents were found to be strongly correlated with the content of most compounds (Figure 6b). The green module, comprising 2687 genes, exhibited a positive correlation with all compounds except hederagenin. In contrast, the blue module, which contains 3673 genes, demonstrated a negative correlation with all compounds, with the exception of pterocaryoside A (Figure 6b,c). The number of TFs in each module ranged from 3 to 203, and four modules (blue, brown, green and yellow) contained more than 100 TFs. The green and blue modules contained 122 and 203 TFs, respectively (Figure 6d).

**Figure A1:**
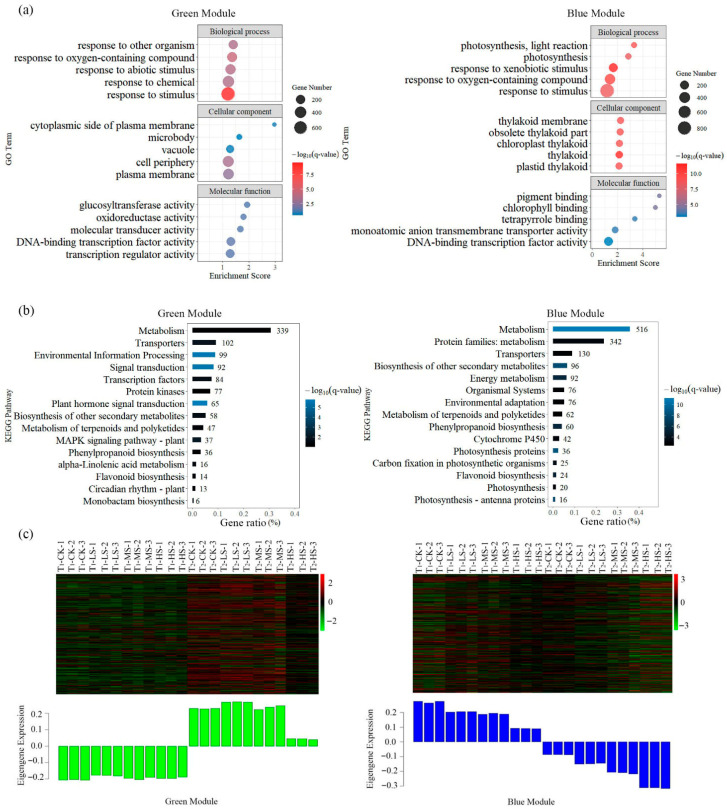
GO (**a**) and KEGG (**b**) enrichment analysis of genes in green and blue modules. (**c**) Eigengene expression profiles of green module and blue module.

To further explore the functional roles of genes in the green and blue modules, GO and KEGG enrichment analyses were conducted (Figure A1a,b). GO results revealed that genes in the green module were enriched in response to abiotic stimulus, while genes in the blue module were enriched in photosynthesis. KEGG results showed that genes in the green module were mainly enriched in signal pathways (such as environmental information processing, signal transduction and plant hormone signal transduction) and secondary metabolism (biosynthesis of other secondary metabolites, flavonoid and metabolism). Conversely, the genes in the blue module exhibited enrichment in carbon metabolism (photosynthesis, photosynthesis proteins and metabolism of terpenoids and polyketides) and secondary metabolism (metabolism of terpenoids and polyketides and biosynthesis of other secondary metabolites). Notably, the expression of eigengenes in the blue module decreased with increasing salt concentration, whereas those in the green module remained relatively unchanged (Figure A1b).

**Figure A2:**
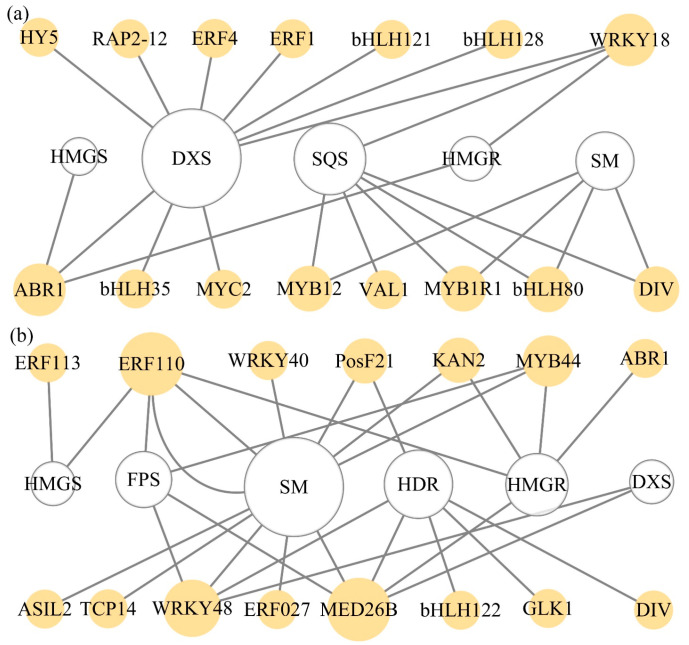
Construction of gene co-expression networks by structural genes with the strongest correlation of TFs. The networks constructed by green (**a**) and blue (**b**) module genes, which were highly correlated on the accumulation of triterpenoid. The white circles represent key structural genes; the yellow circles represent TFs in the module, which become larger as the connection degree increases.

In order to obtain the key regulatory genes, the 15 TFs with the highest weight value and the structural genes associated with them were screened from the green and blue modules, respectively, to construct relative association networks (Figure A2a,b). Five key structural genes were identified in the green module: HMGS (*CpaF1st19994*), DXS (*CpaF1st31202*), SQS (*CpaF1st03088*), HMGR (*CpaF1st31841*) and SM (*CpaF1st43482*), which were found to be regulated by these TFs (Figure A2a). In the blue module, seven key structural genes were identified: in addition to the three aforementioned genes HMGS (*CpaF1st28191*), HMGR (*CpaF1st12554*) and DXS (*CpaF1st05443*), four distinct genes including FPS (*CpaF1st07789*), SM (*CpaF1st31394* and *CpaF1st33485*) and HDR (*CpaF1st12271*) were identified (Figure A2b).

### 2.8. Variation in Gene Expression and Metabolite Accumulation for Triterpenoid Metabolism

**Figure 7:**
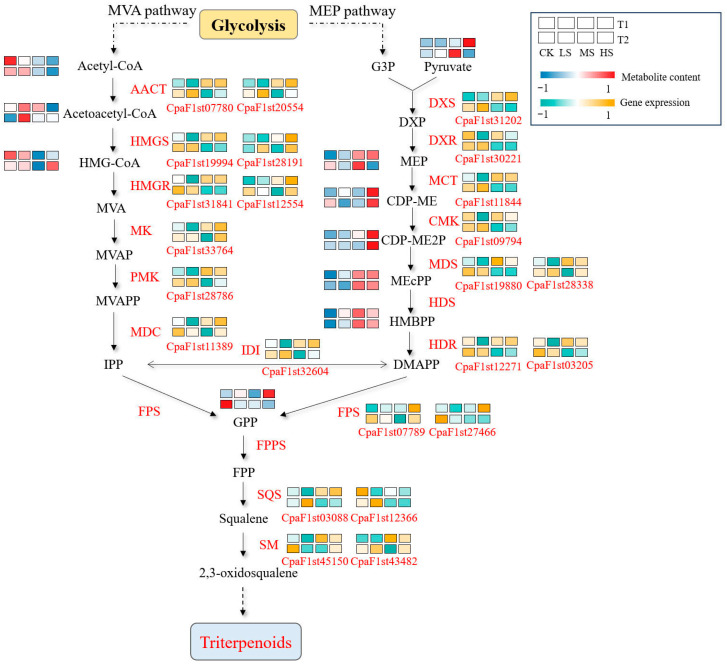
Integrated analysis of the triterpenoid biosynthetic pathway in *C. paliurus*. The green and yellow rectangles represent the heatmap of gene expression. A change from green to yellow indicates gene expression from low to high. The blue and red rectangles represent the heatmap of metabolite abundance. A change from blue to red indicates a change from low to high metabolite content. Refer to Table 1 for abbreviations CK, LS, MS and HS. Metabolites abbreviations: HMG-CoA, (S)-3-hydroxy-3-methylglutaryl-CoA; MVA, mevalonate; MVAP, mevalonate-5P; MVAPP, mevalonate-5PP; IPP, isopentenyl-PP; G3P, D-glyceraldehyde 3-phosphate; DXP, 1-deoxy-D-xylulose 5-phosphate; MEP, 2-C-methyl-D-erythritol 4-phosphate; CDP-ME, 4-(cytidine 5′-diphospho)-2-C-methyl-D-erythritol; CDP-ME2P, 2-phospho-4-(cytidine 5′-diphospho)-2-C-methyl-D-erythritol; MEcPP, 2-C-methyl-D-erythritol 2,4-cyclodiphosphate; HMBPP, 1-hydroxy-2-methyl-2-butenyl 4-diphosphate; DMAPP, dimethylallyl-PP; GPP, geranyl-PP; FPP, farnesyl-PP. Enzyme abbreviations: AACT, acetyl-CoA acetyhransferase; HMGS, 3-hydroxy-3-methylglutatryl-CoA synthase; HMGR, 3-hydroxy-3-methylglutatryl-CoA reductase; MK, mevalonate kinase; PMK phosphomevalonate kinase; MDC, mevalonate pyrophosphate decarboxylase; IDI, isopentenyl-diphosphate Delta-isomerase; DXS, 1-deoxy-D-xylulose-5-phosphate synthase; DXR, 1-deoxy-D-xylulose-5-phosphate reductoisomerase; MCT, 4-diphosphoeytidyl-2-methyl-D-erytbritol synthetase; CMK, 4-diphosphoeytidyl-2-methyl-D-erytbritol kinase; MDS, 2-C-methyl-D-erythritol 2,4-cyclodiphosphate synthase; HDS, (E)-4-hydroxy-3-methylbut-2-enyl-diphosphate synthase; HDR, 4-hydroxy-3-methylbut-2-en-1-yl diphosphate reductas; FPS, farnesyl diphosphate synthase; FPPS, (2Z,6Z)-farnesyl diphosphate synthase; SQS, squalene synthase; SM, squalene monoxydase.

Based on the WGCNA results, a selection of structural genes associated with triterpenoid metabolism were identified within the green and blue modules. The abundance of metabolites and expression of genes in the pathway was mapped based on metabolomic data (Figure 7). The expression of several MVA pathway-related enzymes was found to be upregulated under salt stress, including those encoding AACT (*CpaF1st20554*), HMGS (*CpaF1st28191*), and MK (*CpaF1st33764*), which resulted in an increased accumulation of acetoacetyl-CoA under salt stress. Conversely, acetyl-CoA and HMG-CoA levels increased more significantly in CK. The content of MEP, CDP-ME and CDP-ME2P in the MEP pathway increased with salt concentration, reaching the highest levels in HS. Furthermore, the expression of the encoding genes DXS (*CpaF1st31202*) and MCT (*CpaF1st11844*) was found to be upregulated under salt stress. These results suggest that salt stress may affect the accumulation of triterpenoid metabolites and the activities of related enzymes.

### 2.9. Molecular Docking Analysis of Key TFs and Structural Genes

To further explore the potential reliability of the gene regulatory network constructed by WGCNA, this study employed molecular docking technology to simulate the putative binding conformations and affinities between selected key TFs and their target structural genes involved in triterpenoid biosynthesis. Based on the co-expression network, docking simulations were performed using four TFs with the highest confidence scores predicted by AlphaFold3 and their corresponding structural genes: bHLH35 and MYC2 targeting DXS, ERF113 targeting HMGS, and MED26B targeting HDR.

**Figure 8:**
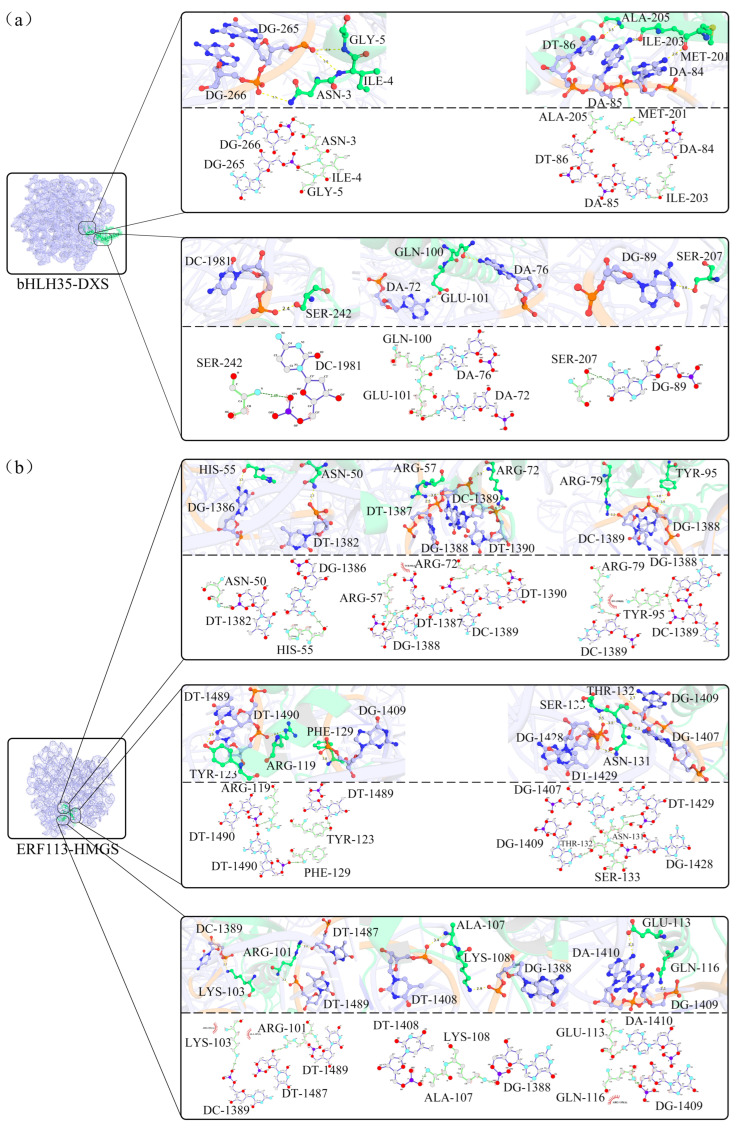
The 3D and 2D structures of molecular docking models of key TFs and their target genes. (**a**) bHLH35 binding to DXS; (**b**) ERF113 binding to HMGS. The left panels display the protein structures predicted by AlphaFold3, while the right panels show magnified detailed views. Above the dotted line in each rectangular panel is the 3D model, and below the dotted line is the 2D representation. Yellow dashed lines in the 3D structures and green dashed lines in the 2D structures represent hydrogen bonds, and the numbers near the dashed lines indicate the hydrogen bond distances.

**Figure A3:**
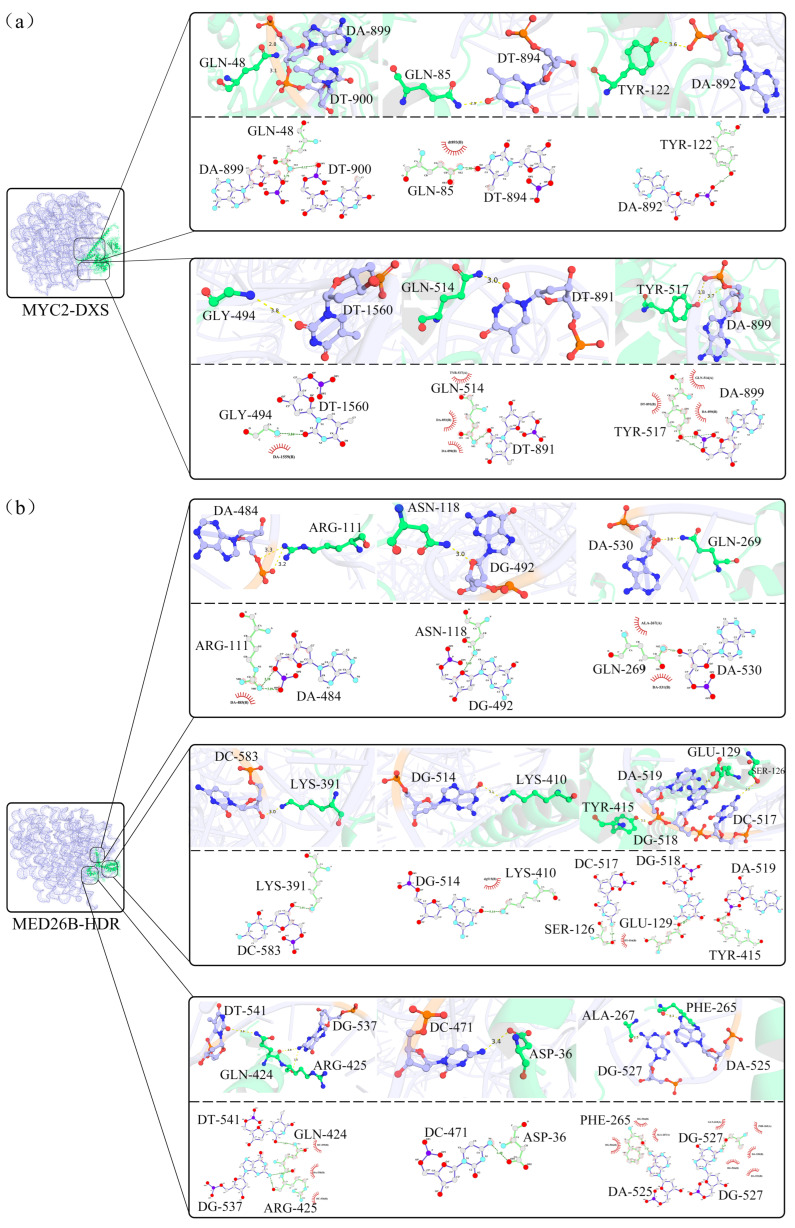
The 3D and 2D structures of molecular docking models of key TFs and their target genes. (**a**) MYC2 binding to DXS; (**b**) MED26B binding to HDR. Refer to Figure 8 for legend details.

The docking results suggested that all four TFs could potentially form stable complexes with the promoter sequences of their respective target genes, a stability that appears to be primarily maintained by dense hydrogen-bond networks (Figure 8 and Figure A3; Table A5). For the key rate-limiting enzyme gene DXS, both bHLH35 and MYC2 exhibited predicted specific binding capabilities, albeit through different interaction modes. bHLH35 was predicted to form approximately 10 hydrogen bonds with DXS nucleotides, relying mainly on residues such as Asn 3, Ile 4, and Ser 242 (Figure 8a). In contrast, the binding of MYC2 to DXS was stabilized by eight putative hydrogen bonds involving glutamine (Gln 48, Gln 85) and tyrosine (Tyr 122, Tyr 517) residues (Figure A3a), suggesting that DXS may be subject to a complex synergistic regulatory mechanism involving multiple TFs.

Notably, the interaction between ERF113 and HMGS exhibited the highest binding density among the tested combinations in silico, forming a predicted network of 25 hydrogen bonds (Figure 8b). The binding interface was enriched with positively charged arginine residues (e.g., Arg 57, Arg 72, Arg 79), which likely promoted strong electrostatic interactions with the DNA backbone. Similarly, MED26B showed high theoretical affinity for HDR, establishing 15 potential hydrogen bonds through key residues such as Arg 111, Lys 391, and Gln 269 (Figure A3b).

## 3. Discussion

Salinity is an undisputed constraint on plant productivity and a potent modulator of secondary metabolism [18,19]. Among metabolic adjustments, triterpenoid accumulation under salt stress has been documented, yet the underlying regulatory circuitry in most woody species remains largely unexplored [20,21]. As a medicinal species, *C. paliurus* is highly valued for abundant triterpenoid compounds in leaves, providing a convincing system to address this gap. The present study aims to elucidate the triterpenoid saponin accumulation pattern in response to salinity and the molecular regulatory network involved.

### 3.1. Salinity Effects on Triterpenoid Accumulation

The optimal defense hypothesis posits that under nitrogen-limiting conditions, plants allocate greater resources to secondary metabolism as a strategy to enhance ecological fitness [22]. As a major class of specialized metabolites, triterpenoid saponins contribute not only to pharmacological applications, but also to stress adaptation. Emerging evidence suggests that certain triterpenoid saponins may actively participate in abiotic stress signaling, thereby reinforcing plant tolerance mechanisms [23,24]. In the present study, salt treatment at T_1_ elicited a significant increase (*p* < 0.05) in total triterpenoid content in *C. paliurus* leaves (Table 2), a pattern consistent with observations in *Glycyrrhiza glabra* under similar conditions [24,25]. At T_2_, although the total triterpenoid content in HS-treated plants was similar to that of the control, the composition changed markedly. For instance, the contents of oleanolic acid, hederagenin, and cyclocaric acid B increased by 196%, 292%, and 53%, respectively, whereas pterocaryoside A and B decreased by 52% and 30%, respectively. This suggests that under prolonged salt stress, the triterpenoid saponins biosynthetic pathway is reprogrammed at specific branch points, leading to a redistribution of metabolic flux toward certain oleanane-type triterpenoids that may play more critical roles in stress adaptation. The unchanged total content likely reflects a dynamic equilibrium between anabolism and catabolism, or between different biosynthetic branches.

Oleanolic acid is widely acknowledged for its multifaceted pharmacological activities, including hepatoprotective, antibacterial and antioxidant properties [26,27]. Notably, oleanolic acid may exhibit analogous biological functions in plants, such as antioxidant properties or a reduction in water loss. Here, a significant increase (*p* < 0.05) in oleanolic acid content was observed under salt treatment at both sampling times in comparison to CK, which is consistent with the findings in *Lantana camara* [28]. Cyclocaric acid, a triterpenoid reportedly unique to *C. paliurus* [29], also exhibited pronounced accumulation in response to salt stress, suggesting a potential adaptive role in osmotic or ionic adjustment. However, not all triterpenoid saponins followed this pattern. Arjunolic acid, for instance, showed no consistent induction, a result that may reflect its inherently low basal abundance in *C. paliurus* leaves and consequent measurement sensitivity constraints, rather than a lack of biological responsiveness. The observations point to compound-specific regulatory mechanisms underlying triterpenoid metabolism under salt stress.

### 3.2. Gene Expression and Metabolite Accumulation in Response to Salt Stress

PCA of the transcriptomic data revealed marked and time-dependent shifts in global gene expression profiles under salt treatments (Figure 2a). Plants exposed to salt stress undergo extensive transcriptional reprogramming, the magnitude and dynamics of which were shaped by stress intensity, duration, and additional interacting factors [13]. Here, the number of DEGs between CK and salt-treated samples increased progressively with rising NaCl concentration (Figure 2b), indicating that high salinity elicited the most pronounced transcriptional restructuring in *C. paliurus*.

Metabolomic PCA similarly demonstrated that both salt concentration and sampling interval significantly influenced metabolite accumulation patterns (Figure 4a). As stress duration extended, the number of DAMs between CK and treatments increased correspondingly (Figure 4b), a trend consistent with observations in other woody perennials under prolonged stress [30]. KEGG enrichment analysis further showed that metabolites differentially accumulated under high salinity, at both time points, were primarily mapped to pathways associated with secondary metabolite biosynthesis (Figure 4c). This enrichment points to a coordinated metabolic defense strategy activated under salt stress, which was also observed in *Cucumis melo* [31].

The Mantel test combined with heatmap analysis of key genes and metabolites revealed that the upregulated genes and metabolites were primarily associated with phenylpropanoid metabolism and the synthesis of secondary metabolites (Figure 5). Furthermore, SOD and CAT activities were strongly correlated with these genes and metabolites, a finding consistent with previous studies on *Eleutherococcus senticosus* [32]. These results suggest that salt-induced accumulation of phenylpropanoids, coupled with enhanced antioxidant enzyme activity, may contribute to improved salt tolerance in *C. paliurus* through more effective scavenging of reactive oxygen species.

### 3.3. Gene Regulatory Networks for Triterpenoid Metabolism

WGCNA is a high-throughput data analysis algorithm based on systems biology principles. It clusters genes with highly similar expression patterns into functional modules and systematically explores biological associations between these modules and phenotypic traits or experimental conditions [33]. This approach is widely adopted to identify transcriptional networks linked to stress tolerance by integrating physiological and transcriptomic datasets [34]. In the present study, two modules (green and blue) exhibiting significant correlations with triterpenoid accumulation were retained for further analysis (Figure 6).

It was reported that WRKY [35,36], bHLH [37,38] and ERF [39,40] play dual roles in modulating triterpenoid biosynthesis and enhancing abiotic stress tolerance Functional characterization of WsWRKY1 in *Withania somnifera* confirmed its positive regulatory effect on triterpenoid accumulation [41]. In apple (*Malus domestica*), overexpression of *MdWRKY18* enhances salt tolerance, while its suppression leads to increased stress sensitivity [42]. Our results showed that, under salt stress, *WRKY18* promoted the expression of genes encoding key enzymes in triterpenoid biosynthesis, including *DXS*, *SQS*, and *HMGR* (Figure A2a). Notably, HMGR and DXS function as rate-limiting enzymes in the mevalonate and methylerythritol phosphate pathways, respectively, while SQS catalyzes the condensation of two farnesyl diphosphate molecules to form squalene [43,44]. Squalene is subsequently converted by squalene monooxygenase into 2,3-oxidosqualene (Figure 7), the pivotal precursor for triterpenoid biosynthesis [45]. These observations suggest a putative role for WRKY18 in contributing to triterpenoid accumulation in *C. paliurus* through the potential transcriptional activation of pathway enzymes.

Similarly, bHLH family members have been implicated in stress responses. Overexpression of *AhbHLH121* from peanut (*Arachis hypogaea* L.) enhances salt tolerance by upregulating antioxidant enzyme activities [46]. In our dataset, *bHLH121* was found to promote the expression of *DXS*, suggesting a potential role in promoting triterpenoid accumulation. Other bHLH members, including *bHLH128* and *bHLH35*, exhibited similar regulatory patterns (Figure A2a). Likewise, ERF TFs contribute to terpenoid metabolism. Overexpression of *GbERF4* isolated from *Ginkgo biloba* and transferred to tobacco (*Nicotiana tabacum*) significantly increased terpenoid content [47]. In this study, *ERF1* and *ERF4* also showed positive correlation with *DXS* expression, thereby increasing triterpenoid accumulation (Figure A2a). Collectively, our findings reinforce the central role of bHLH, WRKY, and ERF TFs in coordinating triterpenoid biosynthesis and salt stress responses in *C. paliurus*.

Despite the identification of MYB12, HY5, GLK1, and several other transcription factors in our co-expression network (Figure A2), their specific roles in regulating triterpenoid metabolism remain largely uncharacterized. Nevertheless, accumulating evidence supports their involvement in the regulation of other secondary metabolites. For instance, ectopic expression of *AtMYB12* from *Arabidopsis thaliana* in *Salvia miltiorrhiza* improved salt tolerance and promoted phenolic acid biosynthesis [48]. HY5 functions as a positive regulator of anthocyanin biosynthesis, and its thermal instability led to reduced anthocyanin accumulation under high temperatures in *A. thaliana* [49]. In addition, GLK1 enhanced the transcriptional activity of *MYB75*, *MYB90*, and *MYB113*, thereby upregulating anthocyanin-specific biosynthetic gene expression in *A. thaliana* [48]. These observations suggest that MYB12, HY5, and GLK1 may similarly participate in triterpenoid regulation in *C. paliurus*, although direct experimental evidence is currently lacking.

Nevertheless, it is important to emphasize that the TFs identified in this study were primarily selected through statistical analyses and in silico molecular docking. While these methods provide a valuable foundation for identifying candidate regulators, they represent computational predictions, rather than direct biochemical evidence. The binding affinities and regulatory interactions inferred here remain predictive in nature. Therefore, further experimental validation is required to gain a more profound understanding of their actual functional roles. Future research should incorporate functional genomics approaches (such as dual-luciferase assays, EMSA, gene overexpression, or CRISPR-based knockout) to verify the regulatory roles of the screened TFs in triterpenoid biosynthesis and stress adaptation in *C. paliurus*.

## 4. Materials and Methods

### 4.1. Plant Materials and Treatments

The experimental material comprised one-year-old, non-flowering seedlings derived from *C. paliurus* seeds (Jinzhongshan No. 11 family, sourced from Guangxi, China) (Figure A1). Seeds were stratified and treated with GA_3_ to break dormancy [50]. Germinated seeds were transferred to nonwoven containers and grown in a perlite: topsoil: manure: peat mixture (2:2:2:4). After three months, uniform seedlings were transplanted to 50 L containers with half-strength Hoagland’s solution (pH 6.0) in a greenhouse at Nanjing Forestry University (31°35′ N, 119°09′ E). Following a two-week acclimatization, four salinity treatments were applied: control (CK, 0 mM NaCl), low (LS, 0.15%), medium (MS, 0.30%), and high (HS, 0.45% *m*/*v*), based on a previous study by Zhang and the salt content of coastal saline surface soil in Jiangsu Province [8,13]. For each treatment, 24 seedlings (50–55 cm height, 3.7–4.2 mm diameter) were used in a completely randomized block design with three biological replicates (*n* = 8). Greenhouse conditions were maintained at 25–32 °C (day) and 17–22 °C (night), with 65–80% relative humidity.

### 4.2. Sample Collection and Measurement of Physiological Indices

Leaf samples were collected at 15 d (T_1_) and 30 d (T_2_) after salt treatment. For each treatment, three seedlings were randomly selected, and six fully expanded leaves per plant were sampled, frozen in liquid nitrogen, and stored at −80 °C.

Plant height and ground diameter were measured for each individual seedling at T_1_ and T_2_. At T_1_ and T_2_, three seedlings were randomly selected from each treatment, and all fresh leaves and roots were collected. The samples were then placed in an oven at 105 °C for 30 min to deactivate enzymes, after which they were dried at 80 °C to constant weight, to obtain the dry weight of leaves and roots.

For biochemical analysis, fresh leaf tissue (0.3 g) was homogenized in phosphate buffer (50 mM, pH 7.0–7.4) containing EDTA and dithiothreitol. After centrifugation (10,000 rpm, 20 min, 4 °C), the supernatant was used to assay superoxide dismutase (SOD), catalase (CAT), and malondialdehyde (MDA) with commercial kits (Nanjing Jiancheng Bioengineering Institute, Nanjing, China) following the manufacturer’s protocols [17].

For ion determination, 2.0 g of dry leaf powder was digested with HNO_3_:HClO_4_ (5:1, *v*/*v*) until clear, diluted to 50 mL with deionized water, and analyzed for K^+^, Ca^2+^, Na^+^, and Mg^2+^ by atomic absorption spectrophotometry (AA-7000, Shimadzu, Kyoto, Japan) [51].

For triterpenoid analysis, 0.8 g of dry leaf powder was ultrasonically extracted with 10 mL of 70% ethanol at 70 °C. Total triterpenoid content was measured colorimetrically. Briefly, the absorbance of the reaction mixture was measured at 540 nm using oleanolic acid as a standard [52]. Individual triterpenoid saponins were analyzed by HPLC (Waters). For the analysis of *C. paliurus* leaves, a binary mobile phase was employed: (A) 0.01% (*v*/*v*) formic acid in water and (B) 0.01% (*v*/*v*) formic acid in acetonitrile. Under a constant flow of 1.0 mL/min and a column temperature of 45 °C, 10.0 μL of sample was injected and monitored at 205 nm. The gradient elution was executed over 100 min: 8–19% B (0–13 min), 19–21% B (13–28 min), 21–50% B (28–42 min), 50% B (42–46 min), 50–55% B (46–60 min), 55–56% B (60–64 min), 56–66% B (64–74 min), 66–85% B (74–90 min), 85–100% B (90–95 min), and 100% B (95–100 min). The system was equilibrated for an additional 15 min after each analysis [53].

### 4.3. Transcriptomic Analysis

Total RNA was isolated using Trizol reagent (Invitrogen, Carlsbad, CA, USA) following Guo [54]. A total of 24 cDNA libraries (four treatments × two time points × three replicates) were constructed and sequenced on an Illumina HiSeq2500 platform (Palo Alto, CA, USA). Clean reads were aligned to the *C. paliurus* reference genome (https://ngdc.cncb.ac.cn/gwh/Assembly/26380/show(accessed on 26 September 2024)) using Hisat2. Transcript abundances were calculated as fragments per kilobase of transcript per million (FPKM). Differentially expressed genes (DEGs) were identified with a false discovery rate (FDR) < 0.05 and |log_2_(fold change)| > 1. All transcriptomic visualizations were produced using R packages implemented in R software (Version 4.4.1) [55]. Principal component analysis (PCA) was conducted using the factoextra package (Version 1.0.7). Volcano plots of DEGs were generated using ggplot2 (Version 3.5.1). A Mantel test combination heatmap was made by linkET package (Version 0.1.0). The trend chart was visualized by Mfuzz (Version 2.64.0).

### 4.4. Metabolomic Analysis

Metabolites were extracted following Guo [54]. Freeze-dried leaf powder (50 mg) was mixed with 1 mL of acetonitrile:methanol:water (2:2:1, *v*/*v*/*v*) containing 0.1 mg/L lidocaine as an internal standard. The mixture was homogenized, sonicated (three cycles), incubated at 20 °C for 1 h, and centrifuged (10,000× *g*, 10 min, 4 °C). The supernatant was filtered and analyzed by UHPLC (1290 Infinity, Agilent, Santa Clara, CA, USA) coupled to a Q Exactive Orbitrap mass spectrometer (Thermo Fisher, Waltham, MA, USA) with a UPLC HSS T3 column. Detection was performed using electrospray ionization (ESI) in both positive- and negative-ion modes. The raw data were converted to mzXML format using ProteoWizard, followed by peak detection, extraction, and alignment using XCMS software (Version 3.2). Metabolite identification was achieved by matching against an in-house database as well as public databases (METLIN). Data were processed against an in-house MS/MS database using OSI-SMMS software (version 1.0). The identified metabolites were subsequently annotated and mapped to the Kyoto Encyclopedia of Genes and Genomes (KEGG) metabolic pathways for functional interpretation. Differentially abundant metabolites (DAMs) were also identified with a FDR < 0.05 and |log_2_(fold change)| > 1.

### 4.5. Weighted Gene Co-Expressed Network Analysis (WGCNA)

**Figure A5:**
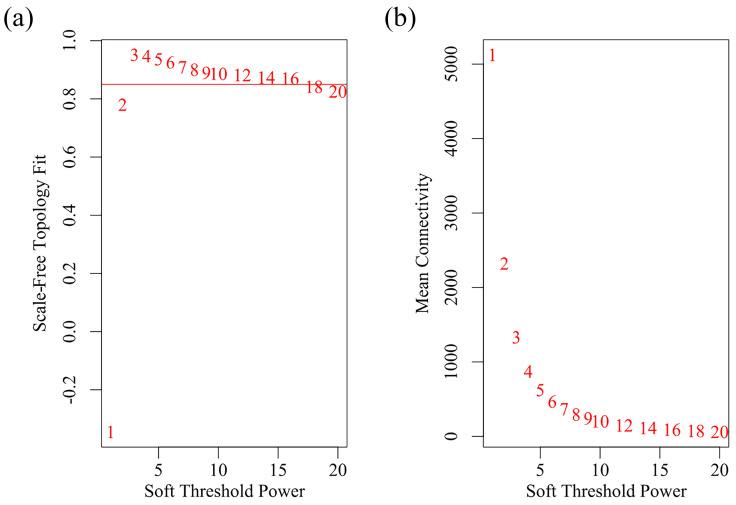
Selection of soft threshold power. (**a**) Plot of scale-free topology fit; (**b**) plot of mean connectivity.

Gene expression data from 24 samples were filtered to retain genes with a coefficient of variation (CV) > 0.5. A weighted gene co-expression network was constructed using the WGCNA package (Version 1.73). To ensure the network conformed to scale-free topology, a soft-thresholding power of 9 was selected, based on the criterion that the scale-free topology fit index (R^2^) reached a threshold of 0.85, combined with the plateauing trend of mean connectivity (Figure A5). Co-expression modules were identified using a dynamic tree-cut algorithm (merge cut height = 0.4, minimum module size = 150). For each module, the module eigengene was calculated and correlated with the concentrations of six triterpenoid saponins. To account for multiple comparisons, *p*-values were adjusted using the Benjamini–Hochberg (FDR) method, and modules with adjusted *p* < 0.05 were retained as significantly trait-associated. Within these modules, transcription factors were ranked by their intramodular connectivity (IC), which quantifies their central role in the module’s regulatory architecture. Genes in these modules were subjected to Gene Ontology (GO) and KEGG enrichment analyses. The top 15 transcription factors with the highest IC and their co-expressed structural genes were identified for further analysis. Regulatory networks were visualized using Cytoscape (Version 3.10.0) [56].

### 4.6. Molecular Docking

Protein structures were predicted using AlphaFold 3 (https://alphafoldserver.com/ (accessed on 15 October 2025)), and protein–DNA docking was performed via the HDOCK server (http://hdock.phys.hust.edu.cn/ (accessed on 23 October 2025)). The resulting complexes were analyzed for interface stability using PDBePISA (https://www.ebi.ac.uk/pdbe/pisa/ (accessed on 8 November 2025)), while 3D conformations and 2D interaction maps were visualized using PyMOL (Version 3.1) and LigPlot^+^ (Version 2.3), respectively.

### 4.7. Statistical Analysis and Visualisations

All statistical analyses and visualizations were performed using OriginPro 2021 (Version 9.8.0.200), SPSS (Version 27.0.1.0), and R software (Version 4.4.1) [55]. GO and KEGG enrichment were analyzed by TBtools software (Version 2.142) [57]. A one-way analysis of variance (ANOVA) was employed to ascertain the disparities between the samples, with significant differences being calculated using the least significant difference (LSD) test at *p* < 0.05. All data in the text are presented as mean ± standard deviation (SD).

## 5. Conclusions

In this study, we employed an integrated transcriptomic and metabolomic approach to investigate the molecular mechanisms underlying triterpenoid biosynthesis in *C. paliurus* leaves under salt stress. Salt treatments significantly altered foliar ion homeostasis, suppressed growth parameters, and induced distinct accumulation patterns of both total and individual triterpenoid saponins. The content of cyclocaric acid B and oleanolic acid exhibited a tendency to increase with increasing salt concentration. Transcriptomic profiling revealed extensive transcriptional reprogramming, with the number of DEGs increasing progressively with stress intensity. WGCNA identified three modules significantly correlated with triterpenoid accumulation, and highlighted several TFs, including WRKY18, bHLH121, ERF4, and ERF1, as potential regulators of key biosynthetic genes such as *DXS*, *SQS*, and *HMGR*. Additional candidates, including MYB12, HY5, and GLK1, were also identified and warrant further functional characterization. The uniqueness of *C. paliurus* lies in its dual properties as both a medicinal and edible plant, as well as a cosmetic and edible resource. Triterpenoid saponins are key active components responsible for its pharmacological functions. Collectively, this work advances the understanding of stress-induced secondary metabolism and offers a theoretical basis for future molecular breeding and genetic improvement of *C. paliurus*, with a view to enhancing triterpenoid saponin production in saline regions.

## Figures and Tables

**Table 1:** Ion content in *C. paliurus* leaves under different salt treatments.

Sampling Time	Treatment	K^+^ Content (mg g^−1^)	Ca^2+^ Content (mg g^−1^)	Na^+^ Content (mg g^−1^)	Mg^2+^ Content (mg g^−1^)
T1	CK	37.35 ± 0.79 c	22.63 ± 0.49 a	0.03 ± 0.02 d	9.59 ± 0.50 a
	LS	37.02 ± 1.51 c	20.53 ± 0.64 b	0.34 ± 0.09 c	7.91 ± 0.47 b
	MS	39.51 ± 1.02 b	18.42 ± 0.72 c	0.70 ± 0.04 b	7.79 ± 0.28 b
	HS	42.24 ± 0.83 a	16.42 ± 0.63 d	1.62 ± 0.15 a	6.83 ± 0.02 c
T2	CK	41.32 ± 1.50 b	23.27 ± 0.46 a	0.26 ± 0.05 c	10.76 ± 0.39 a
	LS	45.51 ± 1.43 a	17.48 ± 0.59 b	0.64 ± 0.08 c	7.75 ± 0.53 b
	MS	45.02 ± 1.81 a	16.82 ± 0.56 b	2.36 ± 0.28 b	7.09 ± 0.17 bc
	HS	47.89 ± 1.13 a	15.76 ± 0.56 c	4.07 ± 0.56 a	6.74 ± 0.26 c

Sampling was conducted at two stages: T_1_ (15 d) and T_2_ (30 d). Salt treatments included CK (control), LS (0.15% NaCl), MS (0.30% NaCl), and HS (0.45% NaCl, *m*/*v*). Data are presented as mean ± SD (*n* = 3); different letters within a column/row indicate significant differences among treatments (*p* < 0.05, Duncan’s test).

**Table 2:** Variations in foliar triterpenoid saponin content of *C. paliurus* under different salt treatments.

Time	Treatments	Triterpenoid Saponin Content (mg g^−1^)						
		Total Triterpenoid Saponins	Arjunolic Acid	Cyclocaric Acid B	Pterocaryoside B	Pterocaryoside A	Hederagenin	Oleanolic Acid
T_1_	CK	31.46 ± 0.57 b	0.11 ± 5.99 × 10^−3^ a	0.16 ± 4.20 × 10^−3^ b	0.24 ± 0.04 d	0.87 ± 0.05 a	0.26 ± 0.03 a	0.11 ± 0.03 c
	LS	36.52 ± 1.39 a	0.07 ±7.09 × 10^−3^ b	0.16 ± 2.08 × 10^−3^ c	0.84 ± 0.02 c	0.25 ± 0.03 c	0.26 ± 0.04 a	0.10 ± 0.02 c
	MS	37.17 ± 2.07 a	0.07 ± 5.54 × 10^−3^ b	0.17 ± 3.39 × 10^−3^ b	1.44 ± 0.04 a	0.44 ± 0.05 b	0.25 ± 0.03 a	0.18 ± 0.04 b
	HS	35.45 ± 2.13 a	0.05 ± 3.37 × 10^−3^ c	0.18 ± 1.22 × 10^−3^ a	1.32 ± 0.04 b	0.20 ± 0.02 c	0.17 ± 0.01 c	0.33 ± 0.05 a
T_2_	CK	43.80 ± 1.08 a	0.10 ± 3.23 × 10^−3^ d	0.30 ± 0.02 c	1.88 ± 0.11 a	0.27 ± 0.02 b	0.13 ± 0.02 b	0.25 ± 0.04 b
	LS	42.15 ± 5.15 a	0.74 ± 0.06 a	0.35 ± 0.01 b	0.74 ± 0.02 c	0.67 ± 0.02 a	0.07 ± 0.01 c	0.56 ± 0.07 a
	MS	39.13 ± 2.32 a	0.26 ± 0.01 b	0.33 ± 0.01 b	1.19 ± 0.04 b	0.69 ± 0.06 a	0.12 ± 0.01 bc	0.61 ± 0.15 a
	HS	43.34 ± 5.84 a	0.19 ± 0.01 c	0.46 ± 0.01 a	1.32 ± 0.10 b	0.13 ± 0.03 c	0.51 ± 0.06 a	0.74 ± 0.12 a

Data are presented as mean ± SD (*n* = 3). Different letters indicate a significant difference (*p* < 0.05) among different salt treatments at each sampling time. Refer to Table 1 for CK, LS, MS, HS, T_1_ and T_2_.

**Figure A4:**
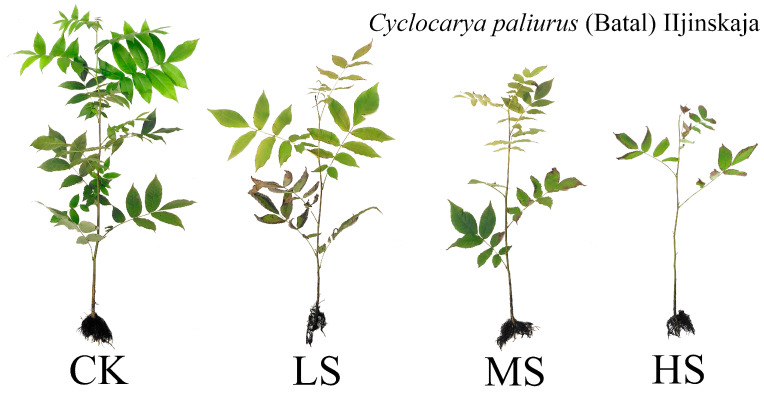
Phenotypes of *C. paliurus* seedlings under different concentrations of salt at T_2_.

## Data Availability

The Illumina raw sequencing profiles were submitted to the NCBI BioProject database under number PRJNA700136.

## Appendix A

**Table A1:** The 39 structural genes related to triterpenoid biosynthesis pathway.

Gene ID	Symbol	Log_2_(FC)					
		T_1_			T_2_		
		LS vs. CK	MS vs. CK	HS vs. CK	LS vs. CK	MS vs. CK	HS vs. CK
*CpaF1st07780*	AACT	−0.88	0.75	0.81	0.35	−1.63 *	−0.47
*CpaF1st20554*	AACT	−0.57	0.82	1.08	0.55 *	−1.50	−0.10
*CpaF1st19994*	HMGS	−0.63	0.22	0.32	0.03	−2.55 *	−1.63 *
*CpaF1st28191*	HMGS	−0.75	0.57	1.32	1.17 *	−0.83	1.30
*CpaF1st02836*	HMGR	−0.98	1.09	−0.12	−1.08 *	−4.02 *	−3.17 *
*CpaF1st12554*	HMGR	0.99	1.59	2.17	−0.44 *	−2.56 *	0.06
*CpaF1st31841*	HMGR	−1.12	0.32	0.32	−0.32	−2.77 *	−2.40 *
*CpaF1st33764*	MK	−0.99	0.32	0.57	−0.04	−1.64 *	0.34
*CpaF1st28786*	PMK	−0.81	0.79	0.63	0.08	−1.04	−0.33
*CpaF1st11389*	MDC	−1.06	0.21	0.49	−0.51 *	−2.54 *	−0.43
*CpaF1st16083*	MDC	−1.00	−0.24	0.31	1.78 *	−0.95	0.19
*CpaF1st05443*	DXS	−1.50	0.20	0.38	−1.73 *	−1.81 *	−2.07 *
*CpaF1st30813*	DXS	−1.31	0.20	−0.19	−0.64 *	−1.6 *	−1.68 *
*CpaF1st31202*	DXS	0.21	0.70	0.86	0.41 *	−1.71 *	−2.27 *
*CpaF1st33309*	DXS	−1.76	−0.31	−0.48	0.32	−1.07	−1.37 *
*CpaF1st43029*	DXS	−1.78	−0.04	−0.43	−0.36	−2.28 *	−1.97 *
*CpaF1st47684*	DXS	−0.91	1.24	0.97	−0.76 *	−2.34 *	−2.38 *
*CpaF1st30221*	DXR	−1.74	−0.18	−0.65	0.12	−2.49 *	−2.63 *
*CpaF1st11844*	MCT	−1.21	0.57	0.47	0.31	−1.84 *	−1.72 *
*CpaF1st09794*	CMK	−1.63	0.04	−0.22	0.20	−2.48 *	−1.38 *
*CpaF1st19880*	MDS	−1.02	0.87	0.33	−0.18	−1.96 *	−2.02 *
*CpaF1st28338*	MDS	−0.96	0.54	0.33	0.23 *	−1.58 *	0.02
*CpaF1st03205*	HDR	−1.58	0.34	0.12	−0.41	−2.99 *	−1.40 *
*CpaF1st12271*	HDR	−1.53	0.08	0.22	−0.18	−1.97 *	−1.17 *
*CpaF1st32604*	IDI	−1.12	0.28	0.48	0.24	−1.56	−0.32
*CpaF1st07789*	FPS	0.60	0.57	1.40	−0.34	−1.63 *	−0.11
*CpaF1st27465*	FPS	−1.75	−0.34	−0.24	−1.92 *	−3.70 *	−1.52 *
*CpaF1st27466*	FPS	−0.80	−0.04	1.00	−1.31 *	−2.01	−2.15 *
*CpaF1st38876*	FPS	−0.89	−0.18	−0.32	−0.28	−2.20 *	−1.60 *
*CpaF1st03088*	SQS	−0.87	0.40	0.59	1.10 *	−0.85	−0.33
*CpaF1st12366*	SQS	−1.52	−0.74	−1.15	0.82 *	−1.46	−1.65 *
*CpaF1st31394*	SM	−0.46	0.82	1.85	−1.03 *	−2.16 *	−0.48
*CpaF1st33485*	SM	−1.77	−0.09	−0.29	−1.29 *	−4.02 *	−2.74 *
*CpaF1st33487*	SM	−2.45	−0.22	−0.45	0.19	−3.52 *	−2.64 *
*CpaF1st43482*	SM	−0.38	2.57	2.00	0.34	−1.47 *	0.09
*CpaF1st45140*	SM	−2.02	1.60	2.04	−4.44 *	−3.47 *	0.61
*CpaF1st45147*	SM	−1.29	0.71	0.75	−0.43 *	−2.35 *	−0.31
*CpaF1st45148*	SM	−2.05	0.05	0.88	−2.73 *	−3.00 *	0.57
*CpaF1st45150*	SM	−3.55	1.04	0.54	−4.35 *	−4.42 *	−0.89

Log_2_(FC) > 1 or <1 indicate significant expression difference between different treatments, * indicates significant differences (*p* < 0.05). Refer to Table 1 for CK, LS, MS, HS, T_1_ and T_2_.

**Table A2:** The 24 metabolites related to triterpenoid biosynthesis pathway.

Metabolite ID	Name	Log_2_(FC)					
		T_1_			T_2_		
		LS vs. CK	MS vs. CK	HS vs. CK	LS vs. CK	MS vs. CK	HS vs. CK
POS12635	Acetyl-CoA	0.44	0.78	−1.55 *	/	/	/
POS12873	Acetyl-CoA	−0.52	−0.20	−1.91 *	−0.39	−4.96 *	−4.29 *
POS13858	Acetyl-CoA	−0.04	−0.52	0.39	0.50 *	−0.96 *	0.14
POS19173	Acetyl-CoA	−0.46	−0.64 *	−0.95 *	−0.25	−0.84	−1.43 *
POS12864	Acetoacetyl-CoA	0.38	0.24	−0.96 *	5.67 *	4.45 *	4.52
POS11156	HMG-CoA	−0.11	−0.59	−0.31	0.10	−2.13	0.50
POS20574	HMG-CoA	/	/	/	3.34 *	/	7.12 *
POS16674	GPP	0.39	−0.48	1.01 *	−1.69 *	−2.56 *	−2.95 *
NEG00039	Pyruvate	1.16	0.11	1.20	−0.83	−1.11 *	−0.81
POS02238	Pyruvate	−0.14	−0.08	0.15	0.28	0.01	−0.06
POS04898	Pyruvate	−0.40	−0.87	−0.53	0.11	0.19	−0.06
POS17935	Pyruvate	−0.01	0.06	0.28	0.10	0.49 *	−0.25
POS17565	MEP	0.11	0.26	0.29	−0.24	0.29	−0.58
NEG06690	CDP-ME	0.37	0.62	−0.12	2.26 *	−1.29	0.80
NEG08732	CDP-ME	0.14	0.04	0.37	−1.88 *	−0.89 *	0.59 *
NEG17267	CDP-ME	−0.01	0.46	−0.20	/	/	/
POS06080	CDP-ME2P	/	/	/	1.4	−0.79	4.79 *
POS09998	CDP-ME2P	2.73 *	2.42 *	2.36 *	1.23	5.20 *	5.29 *
POS11735	CDP-ME2P	0.40	0.60	1.33	/	/	/
POS17907	CDP-ME2P	−1.72 *	−3.77 *	−2.54 *	−0.43	−1.99	−3.79
POS18286	CDP-ME2P	−0.10	0.22	1.27	−0.45	1.40 *	−1.28 *
POS04392	MEcPP	1.24 *	1.84 *	1.84 *	/	3.16 *	3.00 *
POS06995	HMBPP	0.75 *	1.10 *	0.67	1.43 *	1.34 *	1.42 *
POS10272	HMBPP	0.44	0.65	0.49	0.37	0.75 *	0.62

Log_2_(FC) > 1 or <−1 indicate significant expression difference between different treatments, * indicates significant differences (*p* < 0.05). Refer to Table 1 for CK, LS, MS, HS, T_1_ and T_2_. Metabolite abbreviations: HMG-CoA, (S)-3-Hydroxy-3-methylglutaryl-CoA; GPP, Geranyl diphosphate; MEP, 2-C-Methyl-D-erythritol 4-phosphate; CDP-ME, 4-(Cytidine 5′-diphospho)-2-C-methyl-D-erythritol; CDP-ME2P, 2-Phospho-4-(cytidine 5′-diphospho)-2-C-methyl-D-erythritol; MEcPP, 2-C-Methyl-D-erythritol 2,4-cyclodiphosphate; HMBPP, 1-Hydroxy-2-methyl-2-butenyl 4-diphosphate.

**Table A3:** The 20 key genes affecting the growth and development of *C. paliurus*.

Number	Gene ID	Protein Category	Description	Group	*P*_adj_
A	*CpaF1st12957*	F-box/kelch-repeat protein SKIP11	Regulates plant defense metabolic pathways	UP	7.3 × 10^−^^305^
B	*CpaF1st35181*	Aspartate–prephenate aminotransferase	Participates in the tricarboxylic acid cycle and the biosynthesis of aromatic amino acids	UP	1.2 × 10^−292^
C	*CpaF1st00042*	β-carotene hydroxylase	Catalyzes a key step in chloroplast xanthophyll biosynthesis	UP	1.1 × 10^−282^
D	*CpaF1st46329*	Hydroxycinnamoyl -transferase	Plays a role in the synthesis and modification of secondary metabolites	UP	8.0 × 10^−271^
E	*CpaF1st29183*	Carotenoid cleavage dioxygenase	Regulates carotenoid metabolism and influences plant development	DOWN	5.1 × 10^−260^
F	*CpaF1st04301*	Transketolase	Essential for photosynthesis	DOWN	1.2 × 10^−243^
G	*CpaF1st15291*	4-coumarate-coa ligase	Functions in phenylpropanoid metabolism	UP	7.4 × 10^−241^
H	*CpaF1st04909*	ATP sulfurylase	A key enzyme in the sulfate assimilation pathway	DOWN	1.3 × 10^−236^
I	*CpaF1st11918*	Caffeoyl-coa O-methyltransferase	Regulates the biosynthesis of phenylpropenoids and lignin	UP	4.2 × 10^−234^
J	*CpaF1st36188*	Lycopene epsilon cyclase	Catalyzes cyclization reactions in carotenoid biosynthesis	DOWN	2.4 × 10^−228^
K	*CpaF1st13801*	Caffeic acid 3-O-methyltransferase	Contributes to plant secondary metabolic pathways	DOWN	2.2 × 10^−216^
L	*CpaF1st18688*	Cytochrome P450	Involved in the synthesis of secondary metabolites	UP	2.6 × 10^−209^
M	*CpaF1st41336*	β-amyrin synthase	Responsible for oleanane-type triterpenoid saponin biosynthesis	UP	3.4 × 10^−208^
N	*CpaF1st24392*	Leucoanthocyanidin reductase	Catalyzes a step in proanthocyanidin biosynthesis	UP	1.4 × 10^−206^
O	*CpaF1st36377*	Hexokinase	Functions in glycolytic metabolism	UP	3.5 × 10^−205^
P	*CpaF1st38292*	(3S,6E)-nerolidol synthase	Participates in terpenoid biosynthesis	DOWN	4.2 × 10^−204^
Q	*CpaF1st38420*	Cannabidiolic acid synthase	Acts in secondary metabolic pathways	UP	5.6 × 10^−201^
R	*CpaF1st29147*	Abscisic acid 8′-hydroxylase	Negatively regulates abscisic acid levels by catalyzing its oxidative degradation	DOWN	6.4 × 10^−198^
S	*CpaF1st40792*	Crocetin glucosyltransferase	Important for secondary metabolic pathways, regulating pigment biosynthesis	UP	7.9 × 10^−187^
T	*CpaF1st05320*	Primary amine oxidase	Crucial to stress response	UP	3.4 × 10^−186^

**Table A4:** The 10 key metabolites affecting the growth and development of *C. paliurus*.

Number	Metabolite ID	Metabolite Name	Description	Group	*P*_adj_
a	NEG00165	Phenylglyoxylic acid	Involved in phenylalanine metabolism	UP	5.4 × 10^−^^4^
b	NEG00111	Gallic acid	Essential to the biosynthesis of various plant secondary metabolites and phenylpropanoids	UP	1.1 × 10^−3^
c	NEG00102	Secoisolariciresinol	Important for the biosynthesis of various plant secondary metabolites and phenylpropanoids	UP	1.2 × 10^−3^
d	NEG00068	D-Proline	Crucial to arginine and proline metabolism	UP	1.5 × 10^−3^
e	NEG00020	Benzoic acid	Involved in biosynthesis of various alkaloids and secondary metabolites	UP	1.7 × 10^−3^
f	NEG00169	1,3,7-Trimethyluric acid	Involvement in caffeine metabolism	UP	1.7 × 10^−3^
g	NEG00137	Itaconic acid	Essential to C5-Branched dibasic acid metabolism	DOWN	1.9 × 10^−3^
h	NEG00055	Phloretin	Participates in flavonoid biosynthesis	UP	2.1 × 10^−3^
i	NEG00073	Luteolin	Important for flavone and flavonol biosynthesis	UP	2.2 × 10^−3^
j	NEG00074	Galactinol	Involved in galactose metabolism	DOWN	2.2 × 10^−3^

**Table A5:** Information regarding hydrogen bonds between TFs and structural genes.

Group	Number	TFs	Distance (Å)	Structural Genes
bHLH35-DXS	1	Asn 3 (ND2)	3.55	DG 266 (OP1)
	2	Ile 4 (N)	3.8	DG 265 (OP1)
	3	Gly 5 (N)	3.63	DG 265 (OP1)
	4	Ala 205 (N)	3.49	DT 86 (O4)
	5	Ser 242 (OG)	2.45	DC 1981 (OP1)
	6	Glu 101 (OE1)	2.33	DA 72 (N6)
	7	Gln 100 (OE1)	2.42	DA 76 (N6)
	8	Met 201 (O)	2.59	DA 84 (N6)
	9	Ile 203 (O)	2.25	DA 85 (N6)
	10	Ser 207 (OG)	3.79	DG 89 (N1)
MYC2-DXS	1	Gln 48 (NE2)	2.79	DA 899 (O5′)
	2	Gln 48 (NE2)	3.12	DT 900 (OP2)
	3	Gln 85 (NE2)	2.9	DT 894 (O4)
	4	Tyr 122 (OH)	3.63	DA 892 (OP2)
	5	Gly 494 (N)	3.84	DT 1560 (O2)
	6	Gln 514 (NE2)	2.97	DT 891 (O2)
	7	Tyr 517 (OH)	3.69	DA 889 (O5′)
	8	Tyr 517 (OH)	3.82	DA 889 (OP2)
ERF113-HMGS	1	Asn 50 (ND2)	2.53	DT 1382 (OP2)
	2	Arg 57 (NH1)	2.99	DG 1388 (O4′)
	3	Arg 57 (NH1)	2.5	DT 1387 (O3′)
	4	Arg 72 (N)	3.31	DC 1389 (OP1)
	5	Arg 72 (NH1)	2.61	DT 1390 (OP1)
	6	Arg 72 (NH1)	2.57	DT 1390 (OP2)
	7	Arg 79 (NH2)	3.25	DC 1389 (O3′)
	8	Tyr 95 (OH)	3.61	DG 1388 (O3′)
	9	Tyr 95 (OH)	3.77	DC 1389 (OP1)
	10	Arg 101 (N)	3.32	DT 1489 (OP1)
	11	Arg 101 (NH1)	3.35	DT 1487 (O3′)
	12	Lys 103 (NZ)	3.69	DC 1389 (OP2)
	13	Ala 107 (N)	3.43	DT 1408 (OP1)
	14	Lys 108 (NZ)	2.91	DG 1388 (OP1)
	15	Gln 116 (NE2)	2.09	DG 1409 (O5′)
	16	Arg 119 (NE)	3.5	DT 1490 (OP1)
	17	Tyr 123 (OH)	3.86	DT 1489 (O2)
	18	Phe 129 (N)	3.8	DG 1409 (OP1)
	19	Asn 131 (ND2)	3.73	DT 1429 (OP2)
	20	Thr 132 (N)	3.18	DG 1428 (OP1)
	21	Ser 133 (N)	3.54	DG 1428 (OP1)
	22	His 55 (O)	3.49	DG 1386 (N2)
	23	Asn 131 (O)	2.34	DG 1407 (N2)
	24	Thr 132 (OG1)	2.73	DG 1409 (N2)
	25	Glu 113 (OE1)	3.33	DA 1410 (N6)
MED26B-HDR	1	Arg 111 (NH2)	3.28	DA 484 (O5′)
	2	Arg 111 (NH2)	3.19	DA 484 (OP1)
	3	Asn 118 (ND2)	3.05	DG 492 (O4′)
	4	Gln 269 (NE2)	3.81	DA 530 (O3′)
	5	Lys 391 (NZ)	3.01	DC 583 (O3′)
	6	Lys 410 (NZ)	3.14	DG 514 (O6)
	7	Tyr 415 (OH)	2.21	DA 519 (OP1)
	8	Gln 424 (NE2)	3.83	DT 541 (O2)
	9	Asp 36 (OD1)	3.4	DC 471 (N4)
	10	Ser 126 (OG)	3.29	DC 517 (N4)
	11	Glu 129 (OE1)	2.6	DG 518 (N1)
	12	Phe 265 (O)	2.7	DA 525 (N6)
	13	Ala 267 (O)	2.25	DG 527 (N2)
	14	Gln 424 (O)	3.8	DG 537 (N2)
	15	Arg 425 (O)	3.85	DG 537 (N2)

The number following the amino acid residue indicates the position of the amino acid in the protein. The content in brackets represents atoms of amino acid residues.
